# Automatic segmentation of tumour and organs at risk in 3D MRI for cervical cancer radiation therapy with anatomical variations

**DOI:** 10.1007/s13246-024-01415-y

**Published:** 2024-04-24

**Authors:** Sze-Nung Leung, Shekhar S. Chandra, Karen Lim, Tony Young, Lois Holloway, Jason A. Dowling

**Affiliations:** 1 University of Queensland, Brisbane, Australia; 2 CSIRO Australian e-Health Research Centre, Brisbane, Australia; 3 Institute of Medical Physics, University of Sydney, Sydney, Australia; 4 Cancer Therapy Centre, Liverpool Hospital, Sydney, Australia; 5 Liverpool and Macarthur Cancer Therapy Centres and Ingham Institute, Sydney, Australia; 6 South Western Clinical School, University of New South Wales, Sydney, Australia

**Keywords:** Cervical cancer, MRI, Convolutional neural network, 3D multi-organ segmentation

## Abstract

Cervical cancer is a common cancer in women globally, with treatment usually involving radiation therapy (RT). Accurate segmentation for the tumour site and organ-at-risks (OARs) could assist in the reduction of treatment side effects and improve treatment planning efficiency. Cervical cancer Magnetic Resonance Imaging (MRI) segmentation is challenging due to a limited amount of training data available and large inter- and intra- patient shape variation for OARs. The proposed Masked-Net consists of a masked encoder within the 3D U-Net to account for the large shape variation within the dataset, with additional dilated layers added to improve segmentation performance. A new loss function was introduced to consider the bounding box loss during training with the proposed Masked-Net. Transfer learning from a male pelvis MRI data with a similar field of view was included. The approaches were compared to the 3D U-Net which was widely used in MRI image segmentation. The data used consisted of 52 volumes obtained from 23 patients with stage IB to IVB cervical cancer across a maximum of 7 weeks of RT with manually contoured labels including the bladder, cervix, gross tumour volume, uterus and rectum. The model was trained and tested with a 5-fold cross validation. Outcomes were evaluated based on the Dice Similarity Coefficients (DSC), the Hausdorff Distance (HD) and the Mean Surface Distance (MSD). The proposed method accounted for the small dataset, large variations in OAR shape and tumour sizes with an average DSC, HD and MSD for all anatomical structures of 0.790, 30.19mm and 3.15mm respectively.

**Supplementary Information:**

The online version contains supplementary material available at 10.1007/s13246-024-01415-y.

## Introduction

Cervical cancer (CC) is a common disease causing over 300,000 deaths worldwide each year. CC can extend to the pelvic side wall or invade adjacent organs such as the bladder or rectum, and treatment usually involves radiation therapy (RT). Medical imaging is critical for RT treatment planning and Computed Tomography (CT) scans and Magnetic Resonance Imaging (MRI) are generally acquired. MRI provides improved soft tissue contrast and is useful for defining the extent of tumour involvement in terms of contour and volume [1]. External beam RT planning involves some uncertainty caused by organ movements and volume changes, in addition to patient geometric differences. Therefore a margin for the planning dose target volume is generally expanded around the tumour area to ensure it receives adequate radiation dose, resulting in dose being delivered to normal tissues as a side effect [2]. The precise contouring of organ boundaries from medical imaging is essential for accurate treatment application. Conventionally, manual contouring is used, which has limitations including potential inconsistency caused by inter-observer differences, and time inefficiency (the contouring for a single patient requires 90–120 min to be completed [3]). The process also requires specialised staff with a high level of expertise.

For CC, image based automatic segmentation is particularly challenging due to the large inter-patient shape variation and the limited data sources. This may be the main reason for the small number of papers which have explored pelvic organ segmentation for CC treatment planning. Early papers (which did not use deep learning) include Lu, et al. [4] who suggested a unified Bayesian framework for MRI data segmentation resulting in Dice Similarity Coefficient (DSC) values of 0.88, 0.83 and 0.79 for the bladder, uterus and tumour respectively. An alternate approach from Berendsen reported the development of a registration framework for bladder and clinical target volume (CTV) segmentation in MRI [5] with a resulting DSC of 0.73 and 0.57 (median) for the bladder and CTV respectively. Both of these studies used shape prior models and initial planning results as constraints. In a more recent 2021 study [6], a multiatlas-based segmentation model was presented which resulted in a combined DSC of 0.79 for CC organ-at-risks (OARs). These approaches all required prior development of a constraint model which is an inefficient solution with the low accessibility of CC MRI data.

The deep learning approach using a convolutional neural network (CNN) has been explored in recent studies. Most of these models were based on segmenting individual 2D images, ignoring 3D features. The U-Net architecture has been used in a number of studies for CC OARs and tumour segmentation [3, 7]. The use of a 2D and 3D U-Net was tested with segmentation for the gross tumour volume (GTV) from 98 CC diffusion-weighted imaging (DWI) outputs with stage IB to IVA. The resulting DSC ranged from 0.13 to 0.93 with mean and median of 0.77 and 0.83 [8]. In another study [9], the 2D and 3D U-Net was used for the segmentation of 12 anatomical structures from 408 CC CT scans where the segmentation results were used to define the primary CTV. The 2D and 3D model had a mean DSC of 0.81 and 0.82 respectively regardless of inter-observer variability. In a study in 2022, Mask R-CNN was trained for segmentation of the GTV and OARs from 2D MRI with locally advanced CC [10]. The network detected and segmented objects from images provided and assigned a class to the segmented objects. This resulted in a mean DSC of 0.67, 0.70, 0.81 and 0.85 for GTV, uterus, bladder and rectum respectively.

There is currently a gap in the literature related to clinically relevant, fast, 3D segmentation for cervical cancer treatment planning, particularly with limited labelled training data. This paper addresses this gap through the development of a novel network named Masked-Net. This network has been investigated with the use of transfer learning (TL) and applied to CC MRI data. The aim is to provide a 3D approach for segmentation which can provide usable results from the limited sources of MRI data in this domain; while adapting to large shape variations of the anatomical structures (including the tumour volume). The main contributions of this paper include:

1. Fully automated 3D segmentation was validated on a new clinical MRI dataset for female pelvic organs from a previous study [1].
2. A novel network structure was introduced which included additional information for the segmentation process. Crucially this new structure is applicable to CC datasets with small medical imaging datasets with large anatomical structure shape variations.
3. A new loss function was proposed which accounted for the bounding box of the segmentation results, assisting in limiting false positive results. This approach was helpful for target anatomical structures known to occupy a standard region and consisting of one single volume.

## Methods

### Data

Data in this study included 52 volumes (i.e. 2D image series) obtained from 23 adult patients with stage IB-IVB CC with a macroscopic tumour [1]. They were recruited from Liverpool and Macarthur Cancer Therapy Centre, and the Calvary Mater Newcastle and Royal Brisbane Women’s Hospital in Australia. Ethics approval and informed consent was obtained from all patients. MRI volumes were acquired on a 70 cm bore MAGNETOM Skyra 3T scanner during primary RT with a maximum of 7 volumes at different time points per patient resulting in a dataset with a total of 52 labelled image series. Labels for the bladder, cervix, GTV, uterus and rectum were manually contoured by an experienced radiation oncologist (author KL). The MR sequence for each volume was a 2D axial T2-weighted turbo-spin echo with 2 mm slices and a single concatenation [1]. This dataset was obtained in 2013 to 2019 for an inter-observer contour comparison clinical study and has been utilised in this work for the first time for automatic segmentation. The dataset has a wide range of bladder, tumour and rectum sizes with different patient conditions and stage of cancer at the time of scan acquisition, and was selected to investigate the application of deep learning techniques on a small medical imaging dataset with large structural variations.

A set of prostate cancer MRI with a similar field of view has been used in previous studies [11] for automatic segmentation and was used for model transfer learning in this study. The dataset consisted of 211 image series from 38 patients diagnosed with localised prostate cancer [12]. Manual contours were provided for the bladder, body, bone, rectum and prostate.

### Network structure

Different image segmentation approaches were applied on the CC MRI dataset. Semantic segmentation of the target anatomical structures (bladder, cervix, GTV, uterus and rectum) was performed on the dataset and evaluated. For segmentation task applications, the nnU-Net [13] pipeline was used as a current state-of-art. However, for network architecture comparison, the 3D U-Net [14] was applied as a baseline model for performance evaluation. A U-Net consists of contraction layers to condense and extract pattern and information from the input, and a decoder as the reconstruction process for the final result [14]. Skip connections were present to preserve information lost in the encoder part and to be used for the reconstruction process.

**Fig. 1:**
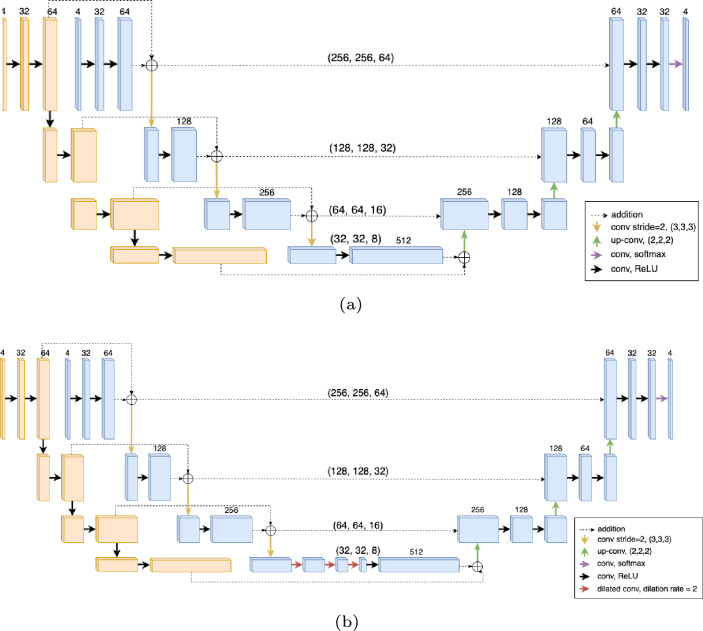
**a** M-Net and **b** DM-Net architecture incorporating additional shape variation information into the neural network training process. Standard convolution blocks include a convolution layer, instance normalisation, a dropout layer with rate of 0.2 and ReLU. Standard dilated convolution blocks include a convolution layer, instance normalisation and Leaky-ReLU. Orange: class label input/ blue: image input

We propose the Masked-Net (M-Net) structure which includes two inputs: manual class labels and MRI image series (see Fig. 1a). The main structure of the M-Net includes an encoder and decoder similar to the 3D U-Net with an additional masked layer for the encoder section. The class labels have the same number of values as the network output, which is the number of target anatomical structures for segmentation. Each channel is manually assigned a class value classifying each anatomical structure based on major shape variations. Convolution and pooling layers are applied on the class label input and added to the main network structure with the corresponding sizes in the encoder section, acting as a mask to the main network, incorporating additional information for the segmentation procedure. Compared to the 3D U-Net [14], the downsampling process was performed with convolution layers with stride of 2 instead of maxpooling, and the skip-connections were included in the decoder section with addition instead of concatenation. The standard convolution block includes a 3D convolution layer, instance normalisation layer, a dropout layer with rate of 0.2 and a ReLU activation layer. These changes were made to increase efficiency during training with the large 3D data size [15].

The addition of dilated convolution layers to the M-Net were also investigated as a modification to the M-Net called DM-Net. The architecture of the network is provided in Fig. 1(b). In contrast to the 3D M-Net, three dilated convolution layers (DCL) [16] were present between the encoder and decoder. The application of dilated convolution was introduced to increase the receptive field of the convolution operation for aggregating contextual information without resolution reduction [11, 16, 17]. Despite its advantages, it has been shown to cause “gridding artefacts" as network outputs are derived from separated input voxels [18, 19]. DCL was used in a previous study for segmentation [11] where the layers were applied consecutively with equal filter numbers and decreasing dilation rates. In this model, DCLs used equal dilation rates with decreasing filter numbers. With the layers applied consecutively, the receptive field was still increased while limiting the gap between voxels in which the output convolution results were obtained. The DCLs were followed by one normal convolution layer and 3 reconstruction layers. Reconstruction layers consist of one up-convolution layer with strides of 2, followed by 2 convolution layers. The up-sampled input of the layer was joined by the output of the contraction layer with corresponding sizes to regain lost information during the encoder part. A last convolution layer with kernel size of 1 and channel size of the number of semantic classes for segmentation was included to obtain the final result. The last layer had an activation function of softmax, which output the probability of each voxel to belong to each output channel.

### Loss function

A new loss function named the Positional Dice Loss (PDL) was also proposed in this work to consider the bounding box differences within the network training process. The combined cross-entropy and Dice loss function (DCE) [20] is widely used for segmentation work. Both the DCE and the Dice loss function ($D_{loss}$) [14] were considered as the basis of the new loss function. Both approaches were tested and compared in Table S2. The DM-Net showed better performance for both the rectum and CGU with the $D_{loss}$ as the basis of the new loss function. As the basis of the PDL, the $D_{loss}$ was used for our model for baseline performance comparison, and the DCE was used to evaluate the performance of the PDL as the current state-of-art. An alternative representation of the $D_{loss}$ was shown as Eq. 1 with p as ground truth and q as predicted results [21]. The new loss function was tested for the training process to learn to identify the region containing the target anatomical structure. It eliminates the inclusion of voxels with similar shapes but which are located far from the actual anatomical structure (i.e. segmentation noise). This was useful for the female pelvis dataset with each anatomical structure known to be a single continuous volume without holes and with its standard location within the female pelvic region. The bounding box difference ($B_{loss}$) was first calculated and added to the $D_{loss}$ to form the PDL (i.e. $PDL = D_{loss} + B_{loss}$). The $B_{loss}$ function is represented in Eq. 2, 3 and 4. Equation 2 outputs the bounding box outline for each axis where equation 3 calculates the sum of the bounding box axis lengths included both the true and predicted results. Both equations were used in equation 4 as the $B_{loss}$.

1 $$\begin{aligned} D_{loss}=1-\frac{2\sum pq}{\sum p^2 + \sum q^2}=\frac{\sum (p-q)^2}{\sum p^2 + \sum q^2}. \end{aligned}$$

2 $$\begin{aligned} S_{x}(X) = \sum _{y,z}X, S_{y}(X) = \sum _{x,z}X, S_{z}(X) = \sum _{x,y}X, S(X) > 0 \rightarrow 1 \end{aligned}$$

3 $$\begin{aligned} R_{x} = count(S_{x}(p+q), S_{x}(p+q) > 0 ) \end{aligned}$$

4 $$\begin{aligned} B_{loss}=\frac{|S_{x}(p) - S_{x}(q)|+|S_{y}(p) - S_{y}(q)|+|S_{z}(p) - S_{z}(q)|}{R_{x}+R_{y}+R_{z}}. \end{aligned}$$

### Implementation

Labelled CC MRI datasets can be difficult to obtain due to the number of organs involved and resources required for manual contouring. With a similar acquisition protocol, prostate MRI data has been widely researched and is relatively more accessible. Therefore our models were pre-trained with a set of prostate cancer MRI with a similar field of view before they were trained with the CC MRI data. The original data size (256x256x128) was cropped to 256x256x64 to fit to our model.

The CC MRI data were divided into 5 sets each including 4–5 patients’ data with a total of 10–11 image series using a 5-fold cross validation. Each of the 5 sets of data was used as the test set once. Four cases from one patient were excluded from the testing sets due to extra large endometrium which was different from other cases within the dataset. While the 4 cases were useful for training, they were not suitable for testing and were identified as outliers. The average and median of the evaluation metric results were obtained to determine the model performances. The entire dataset went through data augmentation 9 times with random operations including elastic deformation, affine warping, uni-axial rotation and shift, resulting in 410–420 image series used for model training within each cross validation set. The patient data included cases where the GTV covered different percentages of the cervix and/or the uterus. To account for these cases, the cervix, GTV and uterus structures were merged for network training purposes. The combined structure (CGU) forms a major portion of the CTV treated with RT. The main reason for combining these structures is that it can be difficult to distinguish anatomical boundaries where the GTV encroaches on different organs. The inclusion of OARs other than the tumour volume are important for generating the treatment dose plan or to identify significant changes in anatomy where dose will need to be re-planned. Data pre-processing included N4 bias field correction [22] and all image series were cropped and resized to the size of 256 x 256 x 64 with a voxel size of 1.64 x 1.64 x 3mm. The class labels for both 3D M-Net and DM-Net were assigned to channel 2 and 3 (bladder and CGU), classifying the target anatomical structures based on bladder size for channel 2 and tumour size for channel 3. The measurements were based on whether the maximum radius of the bladder was above 17 mm for channel 2, and whether the GTV size was larger than 4 times the uterus size for channel 3. Example input volumes are displayed in Figure S1. An example class label input for a case with inflated bladder and large tumour would have a size of 256 x 256 x 64 x 4 with the value of [0, 1, 1, 0] across the volume of the class label input (256 x 256 x 64).

For network training, the Adam optimization algorithm with a learning rate of 0.001 was applied to the model with a batch size of 1 to limit computational cost while optimising model features. The model was implemented on TensorFlow 2.7.0 and the training processes were conducted on a NVIDIA SXM2 Tesla 32 GB V100 GPU. The DSC, Hausdorff Distance (HD) and Mean Surface Distance (MSD) were used as evaluation metrics for the models against the manual contours provided. HD and MSD were reported as DSC is dependent on the volume of the anatomical structures and structure volumes varied significantly across the dataset [23].

## Results

The proposed networks were first tested on the male pelvis dataset used for TL. Results were compared for the bladder, prostate and rectum in terms of mean result DSC, HD and MSD ± standard deviation (SD) in Table S1 and DM-Net demonstrated superior performance with all metrics. Results for the female pelvis dataset were compared to evaluate the model performances with the application of TL, loss functions, and different model structures. Approaches were compared with the average and median of DSC, HD and MSD (mm) for the bladder, CGU and rectum ± SD. To evaluate the impact of TL and the use of PDL for the training process, the standard 3D U-Net was used for comparison (Table 1). U-Net with TL had higher DSC, lower HD and MSD for all three anatomical structures. When trained with PDL, U-Net results were further improved for all three anatomical structures with significant improvement for the CGU results. With TL applied together with PDL, significant improvements were observed for the CGU structure with minor improvements for the remaining organs.

**Table 1:** Evaluation of the new loss function (PDL) and the application of TL

Metric	U-Net		U-Net TL	
	$D_{loss}$	PDL	$D_{loss}$	PDL
Bladder				
DSC	0.810/0.893_±0.193_	**0.819/0.910**_±0.201_	^+^*0.859/0.925*_±0.164_	** 0.868/0.918**_±0.153_
HD (mm)	54.85/48.10_±39.53_	^∗^**41.88/35.52**_±36.01_	^+^*38.62/11.29*_±48.75_	** 31.39/16.03**_±35.14_
MSD (mm)	4.43/2.03_±4.80_	**3.28/1.63**_±3.34_	^+^*2.75/1.35*_±2.80_	**2.56/1.37**_±2.30_
CGU				
DSC	0.608/0.622_±0.154_	^∗^**0.642/0.665**_±0.158_	^+^*0.730/0.773*_±0.139_	^∗^** 0.737/0.776**_±0.140_
HD (mm)	46.89/47.84_±19.81_	^∗^**36.63/38.69**_±15.74_	*41.02/39.46*_±22.00_	^∗^** 38.66/33.71**_±28.57_
MSD (mm)	4.78/4.58_±2.04_	^∗^**4.26/3.95**_±2.21_	^+^*3.58/2.65*_±2.29_	^∗^** 3.42/2.94**_±1.98_
Rectum				
DSC	0.693/0.701_±0.115_	^∗^**0.725/0.744**_±0.105_	^+^*0.777/0.785*_±0.085_	0.769/0.793_±0.092_
HD (mm)	59.39/49.40_±38.29_	**45.88/35.82**_±34.95_	^+^*49.86/27.15*_±48.35_	** 32.78/22.41**_±27.32_
MSD (mm)	3.54/2.60_±2.32_	**3.18/2.49**_±2.31_	^+^*2.79/2.11*_±1.83_	2.83/2.04_±1.99_

The best values are in italic and bold for comparison between loss functions and all methods respectively. + represent metrics with p-value < 0.05 (two-sided paired wilcoxon signed-rank test) with and without TL, with * representing metrics with p-value < 0.05 between loss functions. (mean/median ± SD)

**Fig. 2:**
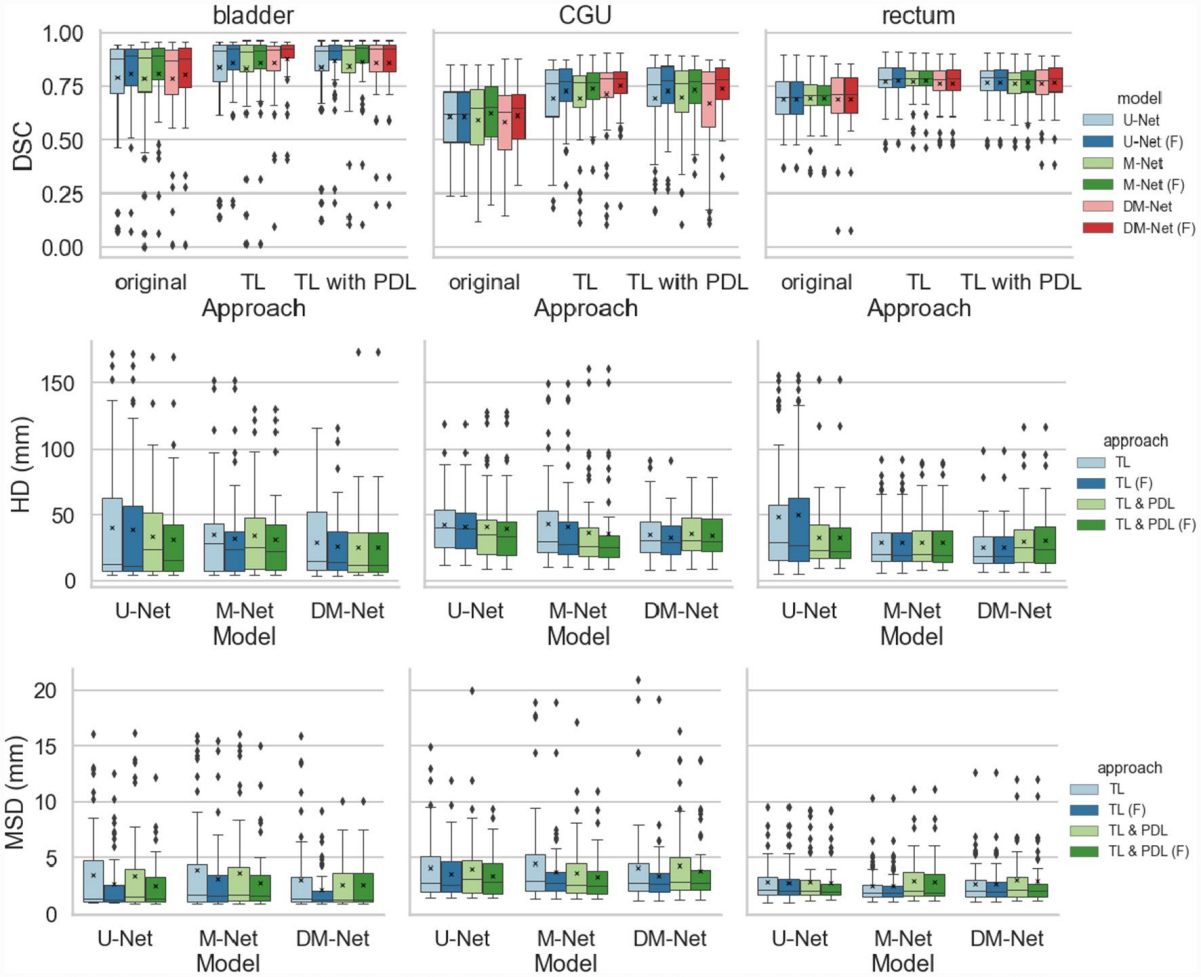
Boxplot of the DSC, HD (mm) and MSD (mm) results for the bladder, combined cervix-GTV-uterus (CGU) and rectum from the proposed approaches. F: dataset without outliers

The results of the proposed networks were compared in Fig. 2 when both TL and PDL were applied to each network approach. Results were displayed for testing sets with and without the outliers in Fig. 2. Comparing the two results, improvements were mainly observed for the bladder and CGU which was expected with the unique uterus appearances (extra large endometria) of the four outliers.

**Fig. 3:**
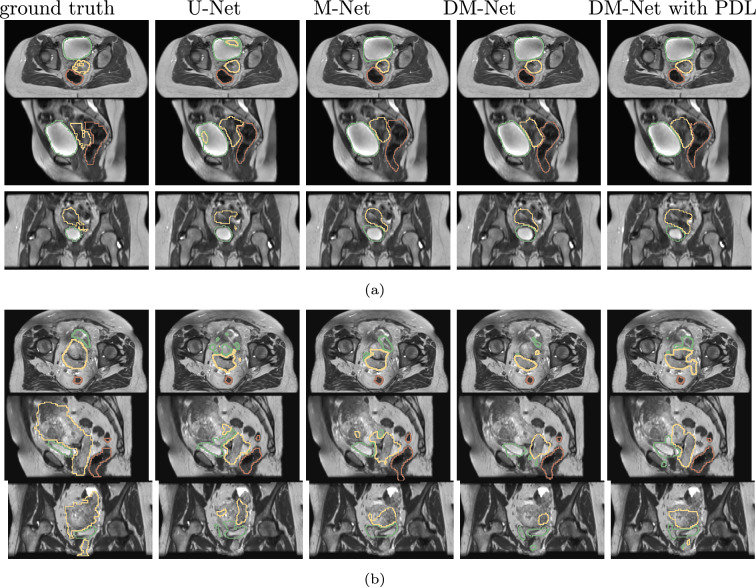
Example results with axial, sagittal and coronal view for networks with TL. **a** Example with average results with inflated bladder and small tumour. **b** Example with small bladder and large tumour with inaccurate results for all approaches. Green: bladder, yellow: CGU, red: rectum

For all anatomical structures and applied approaches, DSC results improved with TL. Improvements in both HD and MSD were seen for most approaches with PDL for the bladder and CGU. However, PDL did not have an obvious impact on DSC results. For all approaches with TL, DM-Net had the best results for the bladder with the best DSC median value and smallest range for both DSC and MSD results. For CGU segmentation, DM-Net had the best overall result in each approach with a smaller range excluding outliers for both the DSC and HD results. For the rectum, the choice of loss function did not have a clinically significant impact on the results. Example outputs are shown in Fig. 3. The displayed patient data results were selected to show the large shape range within the dataset and its effect on each approach. Comparing the application of the DCE and the proposed PDL with the DM-Net (Table S2), the performance of DM-Net with the proposed PDL was superior to using DM-Net with DCE for the rectum and CGU. However, DM-Net with DCE gave the best performance for the bladder.

DM-Net with PDL was compared against the current state-of-the-art nnU-Net framework (Figure S2). When the models were trained for 10 epochs, both DSC and MSD were better with DM-Net and PDL than the nnU-Net. However, the HD results were mixed. To evaluate the approaches’ performance for segmentation applications when optimised, both approaches were trained for 50 epochs with post-processing (removing external contours disconnected to the main body of the semantic labels). Both DM-Net with PDL and nnU-Net established similar metric performances, with higher median DSC results with the nnU-Net.

## Discussion

To demonstrate improvement due to TL with the prostate data, the standard U-Net was trained only with the CC data, and compared with the same network pre-trained with the prostate data for 10 epochs. The pre-trained model weights were transferred into a new model for the CC dataset. All tested approaches were trained with the same CC data. Approaches that used TL all exhibited improved model performance (Table 1). The prostate dataset included contours of the body, bone, bladder, rectum and prostate. It provided extra training data for the model to learn the overall structure of the pelvic region. The model weights trained from the prostate data were transferred into the new U-Net model for CC data training. This allowed a more efficient training process of the model while preventing over-fitting of the available CC data.

**Fig. 4:**
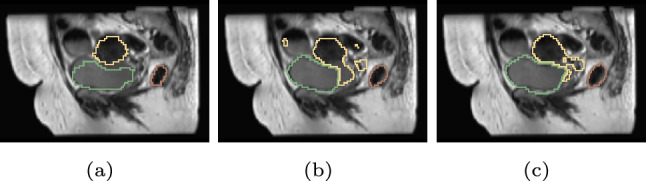
Example result of CGU segmentation (**b**) without or (**c**) with the PDL when TL was applied. Ground truth was displayed in (**a**). Green: bladder, yellow: CGU, red: rectum

Performance with the new loss function was compared with that of the standard 3D U-Net with $D_{loss}$ both with and without TL (Table 1). 3D U-Net trained with PDL without TL improved from the Dice loss application for all anatomical structures where improvements were obvious for the CGU. When PDL was applied with TL, improvement was observed for both the bladder and CGU for all three metrics. For the rectum, the segmentation noise was reduced which was one aim of the proposed approach. An example of the improvement was displayed in Fig. 4. Lack of improvements for the rectum for the DSC and MSD might be caused by its shape. PDL assesses the bounding box of the semantic labels as a cube which did not fit well with the rectum shape. The shape of the bladder was easier to identify compared to CGU, with less noise in the segmentation results. The advantage of PDL was to reduce noise and identify the correct region for the target anatomical structure and this was the likely reason for improved segmentation performance with the CGU compared to the bladder. The DCE is currently often used for segmentation work. However, the PDL had better performance for both the rectum and CGU when applied with the DM-Net (Table S2).

Comparing results obtained from the proposed network structures (Fig. 2), M-Net has the best DSC for the bladder and rectum without TL. However, when TL was applied, DM-Net had the best overall results for both the bladder and CGU. Results were not impacted by different network approaches for the rectum. As only the bladder and CGU were assigned a class value within the class label input of M-Net and DM-Net, the results were expected. DSC did not show the performance gained using PDL, with the three network architectures, as evident with HD and MSD. This indicates that DSC may be less sensitive than HD and MSD as a metric for measuring segmentation noise. However, the effects of PDL were not as obvious for rectum segmentation which was expected as discussed for results from Table 1. It may be more helpful for the segmentation of anatomical structures resembling the shape of a sphere where their bounding box dimensions are more likely to be affected by segmentation errors which was the case for the bladder and CGU compared to the rectum. The M-Net performed better than the U-Net for structures where class labels were given proving the functionality of the masked network. The DM-Net results further improved M-Net and had the best overall DSC score when both TL and PDL were applied, with a higher DSC median and a smaller range. Comparing HD results with or without PDL, the median and range improved with the application of PDL in most cases, proving its ability to reduce noises for segmentation results.

The DM-Net with PDL was compared with the 3D U-Net to investigate its performance with the same data pre-processing and without post-processing applied to the network. Within this study, it provided improvement to the U-Net. When compared with the nnU-Net framework (Figure S2), the proposed approach performed better at a lower epoch number. When trained with increased number of epochs and post-processing, both approaches established similar metric performances with higher median DSC results from the nnU-Net for the bladder and CGU. With the automatic configuration feature of the nnU-Net framework, the outcome is reasonable. The results showed a higher convergence rate with the proposed approach. The use of the DM-Net with PDL within the nnU-Net could be investigated in future studies for segmentation improvements.

There have been previous studies on the segmentation of OAR for CC patients with neural networks on both CT scans and MRI. Studies on CT scans [3, 9] mostly included segmentation for the bladder and rectum and achieved DSC values above 0.90 and 0.78 respectively. For a study which included the segmentation of uterus-cervix [9], a median DSC of 0.90 was achieved. However, comparison with those results is unfair due to the different imaging methods. Compared with previous studies on MRI scans, the DM-Net with PDL method had improved results from a registration framework approach [5] (Table S3). Kurata, et al. reported slightly higher DSC results using a deep learning approach within the CGU region (focusing on uterine segmentation) [7]. However, this work had a dataset with at least 2.5 times more images than the dataset used in this paper with higher image resolution and also only included the GTV and uterus for the tumour segmentation. The dataset used was also performed with different patients’ conditions including different uterine disorders which could affect the results. While current results are likely to be improved by the increased dataset size, the main purpose of this work is to handle datasets with limited size which is a common issue for CC data studies. The data used in this work were also from clinical scans from multiple institutions. The scans were acquired with the same (or as close as possible) imaging sequences with documentation of the imaging sequences. However, there may still be slight differences in MRI acquisition (for example, due to coil positioning) which increased the difficulty of the segmentation task. The Mask R-CNN was also trained with a small dataset with 2D images [10]. It performed better for rectum segmentation but our approach had better results for the bladder and CGU region. With limited dataset size, the DM-Net with PDL was able to achieve reasonable results considering the number of factors affecting the model performance (i.e. structure variability, patient conditions, dataset size and inter-scanner differences). The same approaches were applied on the male pelvis dataset used for the TL step, which had a dataset size of 211 3D images. Compared to the 3D U-Net, results improved for all metrics with the use of the DM-Net (Table S1).

As mentioned in the previous section, the dataset used here was part of a previous study [1] with CC patient data. From that study, inter-observer contouring variability of the dataset was investigated with mean DSC of 0.48 for the cervix and below 0.82 for the GTV and uterus which further demonstrates the difficult nature of CC data. Within the present study dataset, the GTV ranged from 11.42 cm^3^ to 712.31 cm^3^ (median 111.7 cm^3^). This wide range in volumes would likely lead to reduced prediction performance e.g. increased standard deviation. The same situation applied to the bladder and rectum where the bladder volume ranged from 30.08 cm^3^ to 721.8 cm^3^ (median 200 cm^3^) and 19.3 cm^3^ to 213.45 cm^3^ (median 57.95 cm^3^) for the rectum. From Fig. 3, results for two input images were displayed for the approaches with TL applied. For a typical patient with inflated bladder and an average tumour size, the results produced by the M-Net and DM-Net, with TL applied, exhibited less noise and closer agreement to the ground truth than results produced by the U-Net (Fig. 3a). For patient cases with anatomy which is significantly different from normal and also from that contained within the training dataset, all approaches failed to produce reasonable segmentation (Fig. 3b). Comparing different approaches, M-Net was able to reduce the errors from the U-Net with results further improved by DM-Net. PDL was able to increase the coverage area for the CGU segmentation in this case and was able to limit the bladder segmentation to a more reasonable region. The segmentation results were better with an inflated bladder and rectum, and in cases where the gross tumour was close to the median size.

The proposed DM-Net was able to improve the results with the large shape variations for bladder and CGU but there were still cases where segmentation could be improved. In this study, binary class values were given to the target anatomical structures as a basic guidance for segmentation. There was a larger range of shape variation for the rectum and only the bladder and CGU were given a binary class value to investigate the DM-Net. For future studies, the classification approach should be investigated for female pelvis datasets or datasets with large shape variation to further improve the results.

## Conclusions

We presented two novel network structures to address the challenge of 3D segmentation from MRI of the female pelvis with limited patient data: M-Net and DM-Net that included additional information helpful for the 3D segmentation of CC MRI. A new loss function was introduced to limit false positives with the assumption that the target anatomical structures consist of a single volume. In addition to augmented images, transfer learning provided extra training data without the risk of over-fitting. The results demonstrate that these networks are able to account for the limited dataset size that is typical for clinical studies (especially CC). Improvements in performance using the proposed network may facilitate adaptive MRI-based RT workflows, thereby potentially reducing radiation toxicities and improving the quality of life of cervix cancer patients.

## Supplementary Information

Below is the link to the electronic supplementary material.

Supplementary file 1 (pdf 842 KB)
