# Supplementary material for: Automatic segmentation of tumour and organs at risk in 3D MRI for cervical cancer radiation therapy with anatomical variations
Source: Phys Eng Sci Med. 2024 Apr 24;47(3):919–28. doi: 10.1007/s13246-024-01415-y (PMC11408394; doi:10.1007/s13246-024-01415-y)
Supplement: Supplementary file 1 — (pdf 842 KB) [file 13246_2024_1415_MOESM1_ESM.pdf]

A Supplementary data

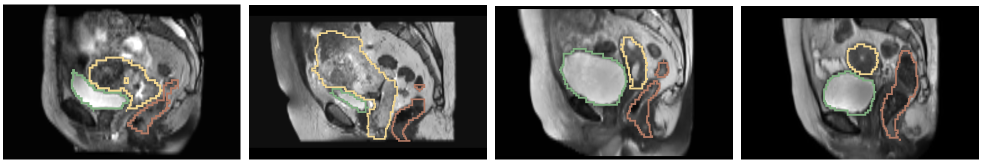

(a) (b) (c) (d)

Fig. S1 Example input images with sagittal view. (a) flat bladder and small tumour. (b) flat bladder and large tumour. (c), (d) inflated bladder and small tumour. Green: bladder, yellow: CGU, red: rectum.

Table S1 Evaluation of the application of proposed networks with the male pelvis dataset. The best values are in bold. (mean±STD)

Region	Metric	U-Net	M-Net	DM-Net
Bladder	DSC	0.907±0.111	0.940±0.043	**0.945**±0.034
	HD (mm)	56.73±64.91	19.16±16.97	**13.04**±14.12
	MSD (mm)	5.86±11.92	1.70±1.60	**1.34**±1.08
Prostate	DSC	0.860±0.047	0.873±0.029	**0.881**±0.032
	HD (mm)	13.45±11.37	11.05±6.33	**10.33**±10.14
	MSD (mm)	1.62±0.76	1.47±0.72	**1.31**±0.27
Rectum	DSC	0.885±0.047	0.881±0.053	**0.903**±0.036
	HD (mm)	23.89±18.28	25.45±25.49	**15.94**±10.05
	MSD (mm)	2.35±1.76	2.63±3.13	**1.88**±1.32

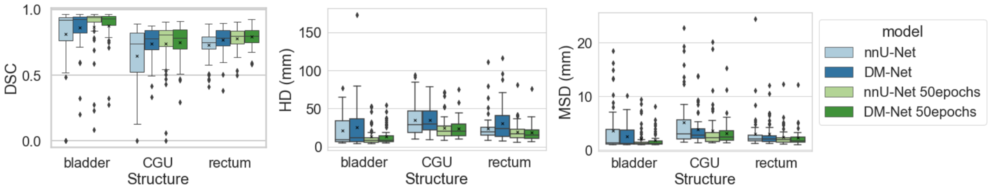

(a) (b) (c)

Fig. S2 Result comparison between nnU-Net and DM-Net with PDL when run with 10 epochs, and 50 epochs with post-processing. Approach performances are compared by (a) DSC, (b) HD (mm) and (c) MSD (mm).

Table S2 Evaluation of the application of the proposed DM-Net with different loss functions. The best values are in bold. (mean/median±STD)

Region	Metric	DCE	DCE and B_{loss}	PDL (D_{loss} + B_{loss})
Bladder	DSC	**0.867/0.927**±0.164	0.861/0.919±0.170	0.861/0.926±0.156
	HD (mm)	**21.22/13.09**±21.51	27.05/11.28±32.67	25.72/11.69±29.14
	MSD (mm)	**2.37/1.45**±2.08	2.80/1.33±2.90	2.60/1.27±2.28
CGU	DSC	0.715/0.756±0.152	0.736/0.780±0.139	**0.740/0.780**±0.129
	HD (mm)	38.63/31.35±31.04	38.32/29.26±29.48	**34.55/30.15**±18.02
	MSD (mm)	4.49/3.15±3.80	4.15/3.01±3.30	**3.87/2.83**±2.95
Rectum	DSC	0.759/0.789±0.102	0.760/0.785±0.109	**0.770/0.786**±0.099
	HD (mm)	32.52/25.01±25.20	40.87/27.91±36.27	**30.31/23.63**±24.16
	MSD (mm)	3.48/2.22±3.87	**2.93/2.06**±2.09	2.98/2.09±2.36

Table S3 Segmentation result DSC comparison with previous studies. The mean and median results were displayed with the minimum and maximum values or ± STD.

**Region**	Berendsen et al.[5] (2013)	Kurata et al.[7] (2019)	Breto et al.[10] (2022)	DM-Net with PDL
Inputs	84 MRI	3350 2D MRI	1245 2D MRI	48 MRI
Patient no.	17	122	15	23
Bladder	0.67/0.73	-	0.81/- ±0.15	0.86/0.93 (0.20-0.97)
CGU	-	-	-	0.74/0.78 (0.33-0.87)
GTV and Uterus	-	0.79/- ±0.065	0.69/- ±0.27	-
CTV	0.55/0.57	-	-	-
Rectum	-	-	0.85/- ±0.09	0.77/0.79 (0.38-0.90)
